# A Discussion on Sensitivity Optimization in Reflective-Mode Phase-Variation Permittivity Sensors Based on Semi-Lumped Resonators

**DOI:** 10.3390/s25030735

**Published:** 2025-01-25

**Authors:** Lijuan Su, Paris Vélez, Pau Casacuberta, Xavier Canalias, Nazmia Kurniawati, Ferran Martín

**Affiliations:** Centre d’Investigació en Metamaterials per a la Innovació en Tecnologíes Electrònica i de Comunicación (CIMITEC), Departament d’Enginyeria Electrònica, Universitat Autònoma de Barcelona, 08193 Bellaterra, Spain; paris.velez@uab.cat (P.V.); pau.casacuberta@uab.cat (P.C.); xavier.canalias@uab.cat (X.C.); nazmia.kurniawati@uab.cat (N.K.); ferran.martin@uab.cat (F.M.)

**Keywords:** microwave sensors, permittivity sensors, phase-variation sensors, step-impedance resonator (SIR), high-*Q* resonators

## Abstract

Typically, the operating frequency in single-frequency reflective-mode phase-variation permittivity sensors based on semi-lumped resonators (e.g., step-impedance resonators—SIRs) is set to the resonance frequency of the sensing resonator when it is loaded with the so-called reference (REF) material, *f*_0,REF_. For the case of an SIR-based sensor, if the ratio between the inductance and the capacitance is high (corresponding to a high-*Q* resonator), the sensitivity in the limit of small perturbations of the dielectric constant (in the vicinity of that of the REF material) is also high. However, the optimum frequency for sensitivity optimization in the limit of small perturbations neither corresponds to the resonance frequency nor coincides with the frequency of maximum phase slope. Such frequencies are calculated in this paper, and it is shown that the optimum frequency for sensitivity optimization is located between the frequency of maximum phase slope and the resonance frequency, although such frequencies tend to coincide for high-*Q* sensing resonators. This aspect is validated in this paper from electromagnetic simulation and experiment.

## 1. Introduction

There are many different approaches for the implementation of planar microwave permittivity sensors, including frequency variation [1,2,3,4,5,6,7,8,9,10,11,12,13,14,15,16,17,18,19,20,21,22,23,24,25,26,27,28,29,30,31,32,33,34,35,36], frequency splitting [37,38,39,40,41,42,43], coupling modulation [44,45,46,47,48,49,50,51,52], and phase variation [53,54,55,56,57,58,59,60,61,62,63,64,65,66,67,68,69,70,71,72,73] (in some sensors, various approaches are exploited simultaneously). Among them, phase-variation sensors are very promising for two main reasons: (i) their robustness against electromagnetic interference (EMI) and noise (as corresponds to phase measurements), and (ii) operation at a single frequency (which represents a reduction in the cost of the associated electronics in a real environment, where vector network analyzers—VNAs—used at laboratory level should be replaced with low-cost components, such as oscillators, gain/phase detectors, etc. [63]).

The specific sensor application determines the target performance parameter. Thus, for wide input dynamic range measurements, sensor linearity is the most important parameter. However, there are many applications where tiny variations in the measurand (input variable) in the vicinity of a reference (REF) value should be resolved and measured. In such cases, a high sensitivity in the limit of small perturbations of the input variable (in the vicinity of that REF value) is required. Thus, a significant research effort has been directed towards the implementation of highly sensitive sensors, and particularly for the design of single-frequency phase-variation permittivity sensors.

It has recently been demonstrated that unprecedented sensitivities can be achieved with one-port reflective-mode phase-variation sensors. Two main strategies or approaches have been considered to boost the sensitivity in such sensors. One such approach utilizes high-*Q* sensing resonators (either distributed or semi-lumped) as the termination of a transmission line [55,58,59,60,61]. As was demonstrated in [55], the sensitivity can be enhanced by cascading a set of high and low impedance inverters, alternating (in practice implemented by means of quarter-wavelength transmission lines) between the input port and the sensing resonator. The second approach is based on weakly coupled sensing resonators, either distributed [58] or semi-lumped [62,64,69]. In this case, very high sensitivities can be achieved, without the need for cascading high/low impedance inverter stages. It has also been demonstrated that, with a proper design, losses benefit sensitivity optimization in reflective-mode phase-variation sensors [65,66]. By contrast, losses tend to degrade the sensitivity in transmission-mode phase-variation sensors [68]. Nevertheless, similar strategies to boost the sensitivity in transmission-mode phase-variation sensors can be applied, particularly cascaded inverters with alternating high and low impedance [68,71], and weakly coupled resonators. It has also been shown that transmission-mode phase-variation sensors exhibiting closely spaced resonance and antiresonance frequencies are effective in regard to sensitivity optimization [70].

The key aspect to achieve a high sensitivity in reflective-mode phase-variation sensors (the type of sensors considered in this paper) is to obtain a high phase slope (in the phase of the reflection coefficient) at the operating frequency when the sensing element is loaded with the REF material (with dielectric constant *ε*_REF_). Since a variation in the dielectric constant of the material under test (MUT) generates a shift in the phase response of the sensor, it is apparent that the excursion experienced by the phase at the operating frequency increases with the phase slope. For this reason, a high phase slope is required for sensitivity optimization. Nevertheless, varying the dielectric constant of the MUT does not generate an overall shift to the left (increasing *ε*_MUT_) or right (decreasing *ε*_MUT_) in the phase response. Rather than that, the phase response is shifted and slightly distorted. Thus, it is not obvious, a priori, that the optimum operating frequency for sensitivity optimization is the one providing the maximum phase slope. This aspect is discussed in this paper by considering a sensor that can be modelled by means of a grounded series resonator (specifically, an SIR-based sensor like the one reported in [61] but excluding the presence of high/low impedance inverters). And this is also the main novelty of this work, demonstrating for the first time that the optimum frequency (*f_opt_*) for sensitivity optimization differs from both the resonance frequency (*f*_0_) and the frequency with maximum phase slope (*f_m_*), i.e., *f*_0_ ˃ *f_opt_* ˃ *f_m_*.

## 2. Analysis

**Figure 1:**
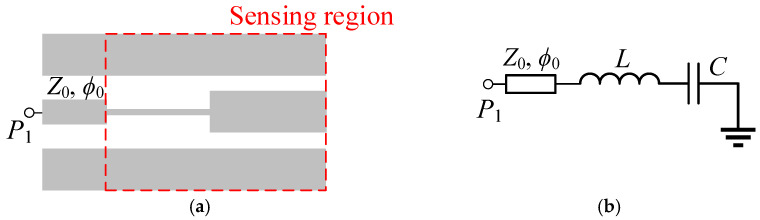
Typical layout (**a**) and circuit model (**b**) of a reflective-mode phase-variation SIR-based sensor (it is considered that the sensor is implemented in CPW technology, but the model is also valid for implementation in microstrip technology).

Let us consider an SIR-based reflective-mode phase-variation sensor that can be described by means of a grounded series resonator (Figure 1). The inductance *L* accounts for the narrow strip of the SIR, whereas the capacitance *C* models the wide strip (or SIR patch). In the layout of Figure 1, a matched access line (with characteristic impedance $Z_{0}$ and electrical length $\phi_{0}$) has been included, but the effects of such a line (an overall phase shift in the phase response) have not been considered in the circuit model. Let us first analyze, according to such a model, the frequency with which the phase of the reflection coefficient exhibits the maximum slope. For that purpose, the first step is to calculate the phase of the reflection coefficient as a function of the angular frequency. The input impedance is

(1) $$Z_{in}=j\left(L\omega -\frac{1}{C\omega }\right)=j\omega L\left(1-\frac{\omega_{0}^{2}}{\omega^{2}}\right)$$

where ω is the angular frequency and $\omega_{0}=1/\sqrt{LC}$ is the angular resonance frequency (i.e., $\omega_{0}=2\pi f_{0}$, $f_{0}$ being the resonance frequency). The reflection coefficient refers to $Z_{0}$, and the reference impedance of the ports (typically $Z_{0}$ = 50 Ω) is

(2) $$\rho =\frac{Z_{in}-Z_{0}}{Z_{in}+Z_{0}}=\frac{jL\omega \left(1-\frac{\omega_{0}^{2}}{\omega^{2}}\right)-Z_{0}}{jL\omega \left(1-\frac{\omega_{0}^{2}}{\omega^{2}}\right)+Z_{0}}$$

Thus, the phase of the reflection coefficient is

(3) $$\phi_{\rho }=2arctan\left\{-\frac{L\omega \left(1-\frac{\omega_{0}^{2}}{\omega^{2}}\right)}{Z_{0}}\right\}+\pi$$

and hence the phase slope is

(4) $$\frac{d\phi_{\rho }}{d\omega }=-\frac{2L}{Z_{0}}\cdot \frac{\left(1+\frac{\omega_{0}^{2}}{\omega^{2}}\right)}{1+{\left\{\frac{L\omega \left(1-\frac{\omega_{0}^{2}}{\omega^{2}}\right)}{Z_{0}}\right\}}^{2}}$$

To find the angular frequency at which the phase slope is at maximum, it is necessary to obtain the derivative of the phase slope and force it to be zero. Such a derivative is

(5) $$\frac{d^{2}\phi_{\rho }}{d\omega^{2}}\;=-\frac{\frac{2L}{Z_{0}}}{D^{2}}\left\{D\left(-2\frac{\omega_{0}^{2}}{\omega^{3}}\right)-2N\left(\frac{L\omega \left(1-\frac{\omega_{0}^{2}}{\omega^{2}}\right)}{Z_{0}}\right)\frac{L}{Z_{0}}\left(1+\frac{\omega_{0}^{2}}{\omega^{2}}\right)\right\}$$

where *D* and *N* are the denominator and numerator, respectively, of the last term in (4). Forcing the previous expression to be zero gives

(6) $$-2\frac{\omega_{0}^{2}}{\omega^{3}}-\frac{2L^{2}\omega_{0}^{2}}{Z_{0}^{2}\omega }{\left(1-\frac{\omega_{0}^{2}}{\omega^{2}}\right)}^{2}-\frac{2L^{2}\omega }{Z_{0}^{2}}{\left(1+\frac{\omega_{0}^{2}}{\omega^{2}}\right)}^{2}\left(1-\frac{\omega_{0}^{2}}{\omega^{2}}\right)=0$$

which is an expression that can be simplified to a biquadratic equation, i.e.,

(7) $${\left(\frac{\omega }{\omega_{0}}\right)}^{4}+2{\left(\frac{\omega }{\omega_{0}}\right)}^{2}+\frac{Z_{0}^{2}}{{L^{2}\omega }_{0}^{2}}-3=0$$

and the solution is

(8) $${\left(\frac{\omega }{\omega_{0}}\right)}^{2}=-1+2\sqrt{1-\frac{Z_{0}^{2}C}{4L}}$$

or (designating the solution as $\omega_{m}$)

(9) $$\omega_{m}=\omega_{0}\sqrt{2\sqrt{1-\frac{Z_{0}^{2}C}{4L}}-1}$$

According to (9), the maximum in the phase slope (or inflexion point in the phase response), $\omega_{m}$, occurs at an angular frequency to the left of $\omega_{0}$. The frequency position of the maximum phase slope depends on the ratio *L*/*C*. If such a ratio satisfies 4 *L*/*C* >> $Z_{0}^{2}$ (corresponding to a high-quality factor of the resonator), then $\omega_{m}\approx \;\omega_{0}$. As the ratio *L*/*C* decreases, $\omega_{m}$ displaces to the left of $\omega_{0}$, and for ratios satisfying *L*/*C* < $Z_{0}^{2}/3$, the main radicand of (9) is negative, and hence there is not a real solution for $\omega_{m}$. This means that for ratios *L*/*C* satisfying the last inequality (*L*/*C* < $Z_{0}^{2}/3$), there is no inflexion point in the phase response, and the maximum phase slope occurs at DC (or $\omega_{m}$= 0).

Let us assume that *L*/*C* > $Z_{0}^{2}/3$, so that there is an inflexion point, $\omega_{m}$, between DC and $\omega_{0}$. This means that for that phase response, dictated by $\omega_{0}$, and the *L*/*C* ratio, the maximum phase slope occurs at that frequency. However, the maximum phase slope at that frequency, $\omega_{m}$, does not necessarily correspond to that phase response. To demonstrate this, let us obtain the derivative of the phase slope (4) with *L* (considering ω and $\omega_{0}$ to be fixed), and let us force it to be zero in order to obtain the optimum value of *L* (and hence the phase response) that maximizes the phase slope. The result is as follows:

(10) $$\frac{d\left(\frac{d\phi_{\rho }}{d\omega }\right)}{dL}=-\frac{\frac{2}{Z_{0}}\left(1+\frac{\omega_{0}^{2}}{\omega^{2}}\right)\left\{1-{\left\{\frac{L\omega \left(1-\frac{\omega_{0}^{2}}{\omega^{2}}\right)}{Z_{0}}\right\}}^{2}\right\}}{{\left[1+{\left\{\frac{L\omega \left(1-\frac{\omega_{0}^{2}}{\omega^{2}}\right)}{Z_{0}}\right\}}^{2}\right]}^{2}}$$

and by forcing it to be zero, one obtains

(11) $$L=\left|\frac{Z_{0}}{\omega \left(1-\frac{\omega_{0}^{2}}{\omega^{2}}\right)}\right|$$

This means that for a given angular frequency, *ω*, the inductance *L* that optimizes the phase slope at that frequency is given by (11) (and the capacitance is thus given by *C* = 1/*L*$\omega_{0}^{2}$). If *ω* = $\omega_{m}$, the value of *L* is not the one corresponding to (9), and therefore, we can conclude that at the frequency of the maximum phase slope for a given phase response, such a phase slope is not the maximum possible value. To gain further insight on this, we have isolated *ω* from (11) and the following result has been obtained:

(12) $$\omega =\omega_{0}\left(\sqrt{1+\frac{Z_{0}^{2}}{{{4L}^{2}\omega }_{0}^{2}}}-\frac{Z_{0}}{2L\omega_{0}}\right)=\omega_{0}\left(\sqrt{1+\frac{Z_{0}^{2}C}{4L}}-\frac{Z_{0}}{2}\sqrt{\frac{C}{L}}\right)$$

This result does not coincide with (9). Note that there is always a positive real solution for (12), whereas this is not the case for (9). Also note that in the limit where 4 *L*/*C* >> $Z_{0}^{2}$, the solution of (12) is $\omega \;\approx {\;\omega }_{0}\;\approx \;\omega_{m}$. In the limit where *L* → ∞, $\omega \;={\;\omega }_{0}=\;\omega_{m}$, the phase slope is maximum at resonance ($\omega_{m}=\;\omega_{0}$), and at that frequency, the phase slope is infinite.

The calculation of the optimum angular frequency for sensitivity optimization was carried out in [61]. Nevertheless, let us reproduce the analysis here for coherence and completeness. Let us suppose that a certain material under test (MUT), with dielectric constant *ε*_MUT_, is placed on top of the SIR. The effect is a variation in the SIR capacitance, i.e., *C′* = *C* + Δ*C*, which in turn modifies the phase of the reflection coefficient, *ϕ_ρ_*. The sensitivity can thus be expressed as

(13) $$S=\frac{d\phi_{\rho }}{d\varepsilon_{\mathrm{MUT}}}=\frac{d\phi_{\rho }}{dC^{\prime }}\cdot \frac{dC^{\prime }}{d\varepsilon_{\mathrm{MUT}}}=\frac{d\phi_{\rho }}{d\Delta C}\cdot \frac{d\Delta C}{d\varepsilon_{\mathrm{MUT}}}$$

Let us next express the input impedance of the SIR when it is loaded with the MUT in terms of the resonance frequency of the unloaded SIR, $\omega_{0}=1/\sqrt{LC}$. The following result is obtained

(14) $$Z_{\mathrm{in}}=j\omega L\left[1-\frac{\omega_{0}^{2}}{\omega^{2}\left(1+\frac{\Delta C}{C}\right)}\right]$$

and, from it, the phase of the reflection coefficient can be expressed as

(15) $$\phi_{\rho }=2\arctan\left\{\frac{\omega L}{Z_{0}}\left[\frac{\omega_{0}^{2}}{\omega^{2}\left(1+\frac{\Delta C}{C}\right)}-1\right]\;\right\}+\pi$$

The first term of the sensitivity, *dϕ_ρ_*/*d*Δ*C*, is found to be

(16) $$\frac{d\phi_{\rho }}{d\Delta C}=\frac{-2}{1+\frac{\omega^{2}L^{2}{\left[1-\frac{\omega_{0}^{2}}{\omega^{2}\left(1+\frac{\Delta C}{C}\right)}\right]}^{2}}{Z_{0}^{2}}}\cdot \frac{L\omega_{0}^{2}}{Z_{0}\omega }\cdot \frac{1/C}{{\left[1+\frac{\Delta C}{C}\right]}^{2}}$$

and such a term in the limit of small perturbations of the dielectric constant of the MUT (or Δ*C* = 0) is

(17) $${\left.\frac{d\phi_{\rho }}{d\Delta C}\right|}_{\Delta C=0}\equiv {\left.S_{\Delta C}\right|}_{\Delta C=0}=\frac{-2Z_{0}L\omega_{0}^{2}/C\omega }{Z_{0}^{2}+L^{2}\omega^{2}{\left(\frac{\omega_{0}^{2}}{\omega^{2}}-1\right)}^{2}}$$

The last term in (13) does not depend on the angular frequency. Thus, to determine the frequency that optimizes the sensitivity, it is necessary to derive (17) with the angular frequency and force the result to be null. The resulting equation is found to be

(18) $$3{\left(\frac{\omega }{\omega_{0}}\right)}^{4}-\left(2-\frac{Z_{0}^{2}}{L^{2}\omega_{0}^{2}}\right){\left(\frac{\omega }{\omega_{0}}\right)}^{2}-1=0\;$$

and the solution is

(19) $${\left(\frac{\omega }{\omega_{0}}\right)}^{2}=\frac{1}{3}-\frac{1}{6}\frac{Z_{0}^{2}}{L^{2}\omega_{0}^{2}}+\frac{2}{3}\sqrt{1+\frac{1}{4}\left(\frac{1}{4}\frac{Z_{0}^{4}}{L^{4}\omega_{0}^{4}}-\frac{Z_{0}^{2}}{L^{2}\omega_{0}^{2}}\right)}$$

or (designating the solution as $\omega_{opt}$)

(20) $$\omega_{opt}=\omega_{0}\sqrt{\frac{1}{3}-\frac{Z_{0}^{2}C}{6L}+\frac{2}{3}\sqrt{1+\frac{1}{4}\left(\frac{Z_{0}^{4}C^{2}}{4L^{2}}-\frac{Z_{0}^{2}C}{L}\right)}}$$

According to (20), the angular frequency where the sensitivity in the limit of small perturbations is optimized and is located to the left of $\omega_{0}$. In the limit when *L*/*C* is very large, $\omega_{opt}=\omega_{0}$, coinciding with the frequency where the phase slope is also a maximum, $\omega_{m}$. However, if the ratio of *L*/*C* is moderate, both $\omega_{opt}\;$ and $\omega_{m}$ are lower than $\omega_{0}$.

Let us next determine whether $\omega_{opt}\;$> $\omega_{m}$, or vice versa. For that purpose, and because the interest in highly sensitive reflective-mode phase-variation sensors is to use high-*Q* resonators (providing a high phase slope in the vicinity of resonance), and hence 4 *L*/*C* >> $Z_{0}^{2}$, let us approximate the inner square roots in (9) and (20) by their respective first-order Taylor series approximations. For (9), the result is

(21) $$\omega_{m}=\omega_{0}\sqrt{2\left(1-\frac{Z_{0}^{2}C}{8L}\right)-1}=\omega_{0}\sqrt{1-\frac{Z_{0}^{2}C}{4L}}$$

whereas for (20), one obtains

(22) $$\omega_{opt}=\omega_{0}\sqrt{\frac{1}{3}-\frac{Z_{0}^{2}C}{6L}+\frac{2}{3}\left(1+\frac{1}{8}\left(\frac{Z_{0}^{4}C^{2}}{4L^{2}}-\frac{Z_{0}^{2}C}{L}\right)\right)}=\omega_{0}\sqrt{1-\frac{Z_{0}^{2}C}{4L}+\frac{Z_{0}^{4}C^{2}}{4{8L}^{2}}}$$

and it is apparent that ${\omega_{0}>\omega }_{opt}\;$> $\omega_{m}$.

## 3. Validation

For validation of the previous analysis, let us consider two different sensing structures consisting of reflective-mode phase-variation sensors based on SIRs and implemented in CPW technology. The difference concerns the ratio *L*/*C*, whereas the resonance frequencies of the bare sensors are identical in both cases. Such a frequency is set to ${f_{0}=\omega }_{0}/2\pi \;=1/2\pi \sqrt{LC}$ = 2 GHz. In the sensor designated by A, the above cited ratio is set to satisfy $\sqrt{L/C}=\;$36.4 Ω. With this resonance frequency and *L*/*C* ratio, the inductance and the capacitance of the SIR are found to be *L* = 2.90 nH and *C* = 2.18 pF, respectively, whereas the angular frequency of the maximum phase slope is [according to expression (9)] $\omega_{m}=0.6741\omega_{0}$ (or ${f_{m}=\omega }_{m}/2\pi \;$= 1.3482 GHz). On the other hand, the angular frequency that optimizes the sensitivity is [using (20)] $\omega_{opt}={0.7726\omega }_{0}$, or $f_{opt}=\omega_{opt}/2\pi \;$= 1.5452 GHz. For this case (Sensor A), the ratio *L*/*C* is comparable to $Z_{0}^{2}$, and, for that reason, $\omega_{0},\;\omega_{opt},$ and $\omega_{m}$ are substantially different (and, of course, $\omega_{0}>\omega_{opt}>\omega_{m}$, as predicted in the previous section).

For Sensor B, we set $\sqrt{L/C}=$ 95.4 Ω. The inductance and capacitance of the SIR in this case are *L* = 7.59 nH and *C* = 0.83 pF, respectively, whereas the angular frequency of maximum phase slope is [according to expression (9)] $\omega_{m}=0.9644\omega_{0}$ (or ${f_{m}=\omega }_{m}/2\pi \;$= 1.9289 GHz). Using (20), the optimum angular frequency for sensitivity optimization is found to be $\omega_{opt}={0.9657\omega }_{0}$, or $f_{opt}=\omega_{opt}/2\pi \;$= 1.9314 GHz. Since for Sensor B the ratio *L*/*C* is significantly larger than $Z_{0}^{2}$, both $\omega_{opt}$ and $\omega_{m}$ are very close to $\omega_{0}$, and also, in this case,${\;\omega }_{0}>\omega_{opt}\;$> $\omega_{m}$, as expected. Nevertheless, for Sensor B, it is reasonable to consider that the optimum frequency for sensitivity optimization is the resonance frequency, provided $\omega_{0}\approx \omega_{opt}\approx \omega_{m}$.

**Figure 2:**
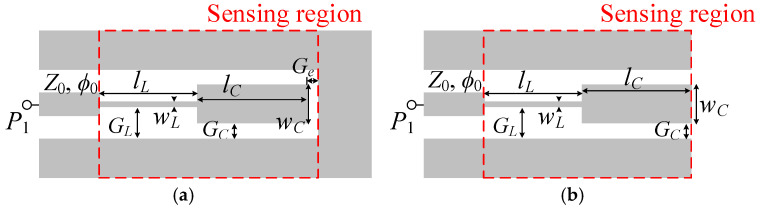
Layout of Sensor A (**a**) and Sensor B (**b**). Dimensions for Sensor A (in mm) are *w_L_* = 0.29, *G_L_* = 3.135, *l_L_*= 2.3, *w_C_* = 6.16, *G_C_* = 0.2, *G_e_* = 0.2, and *l_C_* = 4.67. For Sensor B, they are (in mm) *w_L_* = 0.2, *G_L_* = 3, *l_L_*= 8.6, *w_C_* = 5.2, *G_C_* = 0.5, and *l_C_* = 6.

Let us next synthesize these sensors. The substrate considered for Sensor A is the *Rogers RO3010* with dielectric constant *ε_r_* = 11.2, thickness *h* = 1.27 mm, and loss factor tanδ = 0.0023. The substrate considered for Sensor B is the *Rogers RO4003C* with dielectric constant *ε_r_* = 3.55, thickness *h* = 1.524 mm, and loss factor tanδ = 0.0022. With such low loss factors, losses can be neglected to a first-order approximation. The reason to implement Sensor A in a substrate with higher dielectric constant is because a large equivalent capacitance, *C*, should be generated in order to achieve a lower quality factor (corresponding to a small *L*/*C* ratio). Apart from the difference in the considered substrate, the layout of Sensor A (Figure 2a) is modified from that of the typical layout [shown in Figure 1a]. Specifically, in Sensor A, the ground planes are connected (so that the SIR patch is surrounded by the ground plane). By this means, a larger SIR capacitance, *C*, can be obtained. The dimensions for Sensor A and Sensor B are given in the caption of Figure 2. The synthesis of each sensor is simple, since the inductance is determined by the length of the narrow strip of the SIR, whereas the capacitance is controlled by the width of the wide strip.

**Figure 3:**
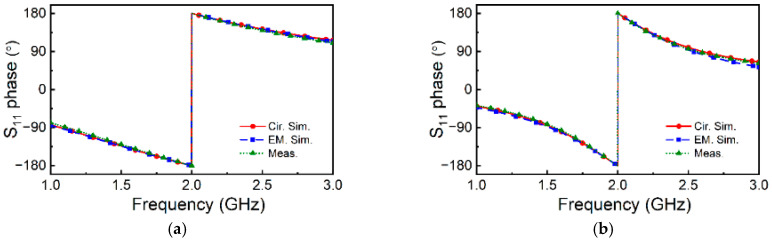

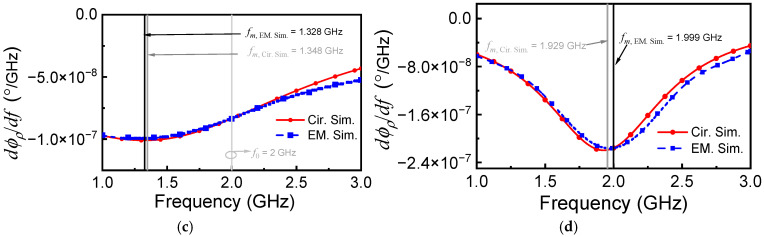
Phase response of Sensor A (**a**) and Sensor B (**b**), and derivative of the phase response for Sensor A (**c**) and Sensor B (**d**).

**Figure 4:**
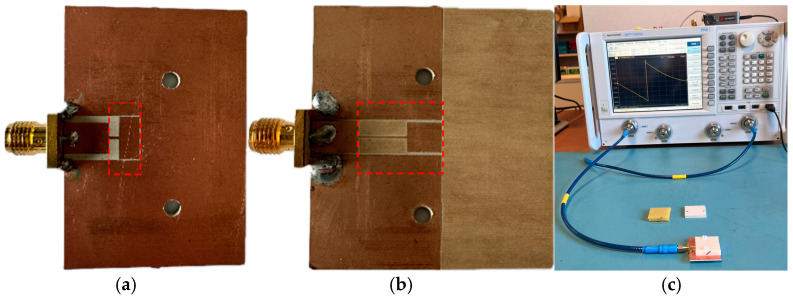
Photograph of the fabricated Sensors A (**a**) and B (**b**), and the measurement setup (**c**).

The phase response of both sensors, inferred from full-wave electromagnetic simulation (using *CST Microwave Studio* commercial software) and measurement (using *Keysight 5221A* vector network analyzer), is depicted in Figure 3 (the fabricated sensors are depicted in Figure 4). The Figure also includes the phase of the reflection coefficient inferred from the circuit simulation (using *Keysight ADS*), with the above-indicated *L* and *C* values of the SIR for each case. The agreement is excellent, and the phase slope is optimized at the indicated frequencies, which coincide with the predictions of the model to a very good approximation. To demonstrate this aspect, the derivative of the phase response is depicted for both structures in Figure 3c,d. The maximum slope of the phase for Sensor A is at *f_m_*_, EM. Sim._ = 1.328 GHz and *f_m_*_, Cir. Sim._ = 1.348 GHz, for electromagnetic simulation and circuit simulation, respectively. For Sensor B, we obtained *f_m_*_, EM. Sim._ = 1.999 GHz and *f_m_*_, Cir. Sim._ = 1.929 GHz for electromagnetic simulation and circuit simulation, respectively.

**Figure 5:**
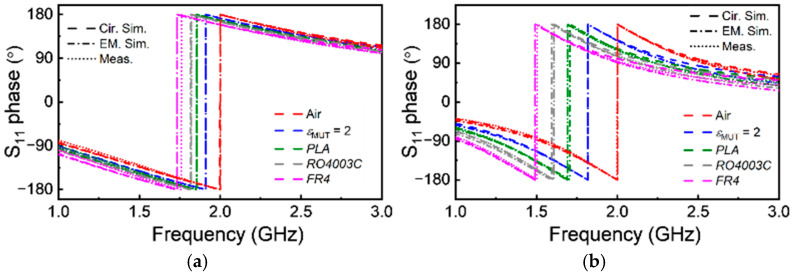
Phase responses of Sensors A (**a**) and B (**b**) when the sensing region is loaded with MUTs of different dielectric constants. The dielectric constant of the loaded MUTs is *ε*_MUT_ = 2, *ε*_PLA_ = 2.7, *ε*_RO4003C_ = 3.55, and *ε*_FR4_ = 4.5. The thickness of MUTs is set to 4 mm, which is enough to satisfy the semi-infinite approximation. Measured data for *ε*_MUT_ = 2 are not provided, since we do not have a sample with this dielectric constant value. The extracted capacitances for Sensor A with the different MUTs loading on the top are (in pF) *Cε*_MUT = 2_ = 2.44, *Cε*_PLA_ = 2.60, *Cε*_RO4003C_ = 2.69, *Cε*_FR4_ = 2.98; for Sensor B, the extracted capacitances are (in pF) *Cε*_MUT = 2_ = 1.02, *Cε*_PLA_ = 1.18, *Cε*_RO4003C_ = 1.31, *Cε*_FR4_ = 1.52. The inductance values for Sensors A and B do not vary with the presence of the MUT, and the values are *L* = 2.90 nH and *L* = 7.59 nH for Sensors A and B, respectively. The EM simulation and circuit simulation were conducted in lossless.

Next, we simulated the phase responses of Sensors A and B by considering the sensing region (indicated in Figure 2) loaded with semi-infinite MUTs of varying dielectric constant in the vicinity of that of air, the reference dielectric constant of the considered sensors. Such phase responses, depicted in Figure 5, reveal that, as the dielectric constant of the MUT increases, the resonance frequency (indicated by the phase jump) displaces to the left, as expected. Figure 5 also includes the measured responses, as well as the circuit responses with extracted parameters. The measured responses were obtained using VNA *Keysight 5221A*, with the measurement setup depicted in Figure 4c. The VNA source power was set to its default value (0 dBm), and a one-port calibration was performed using a *Keysight N7554A* electronic calibration module. Port extension was carried out with open-short-load stubs to align the reference plane of the measurements with the input port. The measurements were conducted under ambient conditions, with a room temperature of approximately 22 °C and a relative humidity of 36%.

**Figure 6:**
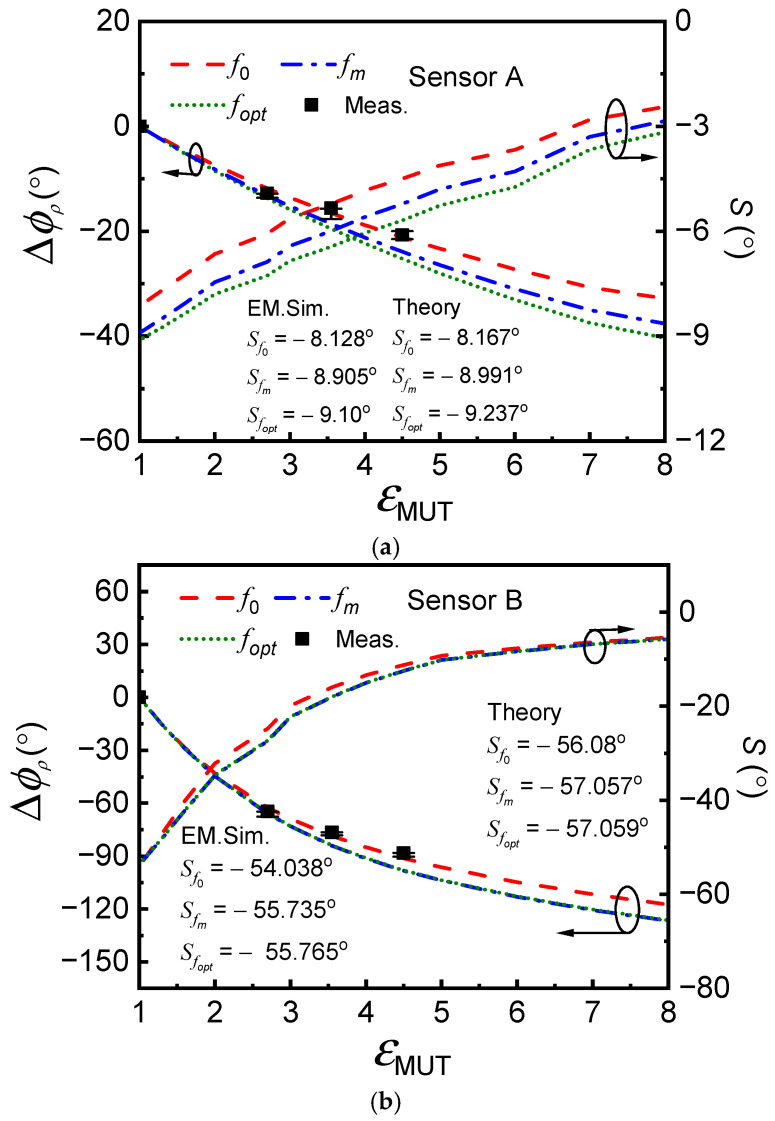
Phase of the reflection coefficient as a function of the dielectric constant of the MUT at the indicated frequencies for Sensors A (**a**) and B (**b**), and sensitivity. The MUTs for measurements are *ε*_PLA_ = 2.7, *ε*_RO4003C_ = 3.55, and *ε*_FR4_ = 4.5. Note that the measured data are plotted at resonant frequency *f*_0_. The measurements were repeated three times for each sample, and the error bar is included in the Figure.

The variation in the phase of the reflection coefficient with *ε*_MUT_ for the specific frequencies *f*_0_, *f_m_*, and *f_opt_* for each sensor are depicted in Figure 6. The Figure also includes the sensitivity for each case, as inferred from the derivative of the phase of the reflection coefficient at the considered frequency with the dielectric constant of the MUT. The sensitivity in the limit of small perturbations exhibits a larger value for *f_opt_*, in agreement with the analysis carried out in the previous section. The difference is more significant in Sensor A, where the separation between *f*_0_, *f_m_*, and *f_opt_* is higher. Nevertheless, the sensitivity in the limit of small perturbations by considering sensor operation at *f_opt_* is superior in Sensor B, because the ratio *L*/*C* is larger in that sensor.

The values of the sensitivities in the limit of small perturbations for operation at the considered frequencies obtained from the simulated data points can be confronted with the results inferred from the theory. For that purpose, the first term of the sensitivity (13), given by (17), should be used by replacing the angular frequency, ω, with the specific considered angular frequency, either ${\;\omega }_{0},\omega_{opt},\;or\;\omega_{m}$. As for the second term, under the semi-infinite MUT approximation considered, it is given by [61]

(23) $$\frac{d\Delta C}{d\varepsilon_{\mathrm{MUT}}}=\frac{C}{\varepsilon_{r,\mathrm{eq}}+1}$$

where $\varepsilon_{r,\mathrm{eq}}$ is the equivalent dielectric constant of the substrate, defined as the dielectric constant of a hypothetical semi-infinite substrate providing the same contribution to the capacitance of the resonant element. Such an equivalent dielectric constant can be calculated according to the method reported in [74], based on electromagnetic simulations (using *ANSYS HFSS* Electromagnetics Suite 16.2.0.). The value has been found to be $\varepsilon_{r,\mathrm{eq}}$ = 9.22 for *Rogers RO3010*, and $\varepsilon_{r,\mathrm{eq}}$ = 2.9 for *Rogers RO4003C*, i.e., somehow smaller than $\varepsilon_{r}$, the nominal dielectric constant of the considered substrates. Using (17) and (23), and introducing the results in (13), the theoretical sensitivities at the considered frequencies are inferred. The results are also indicated in Figure 6, where it can be appreciated that the agreement with the results inferred from the simulations is good in all cases.

Obtaining the sensitivity in the limit of small perturbations with certain accuracy experimentally is not possible due to the lack of a significant number of available materials with a dielectric constant close to that of air (the REF dielectric constant). Nevertheless, we have obtained the phase of the reflection coefficient at the frequencies considered for different MUTs, as indicated in the caption of Figure 5. In more detail, such MUTs are stacks of uncladded microwave substrates available in our laboratory, specifically, *FR4* ($\varepsilon_{MUT}$ = 4.5) and *Rogers RO4003C* ($\varepsilon_{MUT}$ = 3.55). The estimated thickness of the MUT stacks is roughly 4.5 mm, sufficient to consider the MUTs semi-infinite. Nevertheless, we have also considered a piece of polylactic acid (PLA), with thickness 4 mm, fabricated by means of a 3D-printer (the *Ultimaker 3 Extended*), with dielectric constant $\varepsilon_{MUT}$ = 2.7. The measured phases are included in Figure 6, with good agreement with the simulated values results. Note that the sensitivity in the limit of small perturbations is higher in Sensor B (regardless of the specific considered operational frequency), but linearity is better in Sensor A. Because the linearity in Sensor A is relatively good, the differences in the sensitivities (in the limit of small perturbations) for sensor operation at the three considered frequencies are not very important, despite the fact that such frequencies are very different for that sensor. For Sensor B, the linearity is not as good as for Sensor A, but the fact that the three frequencies are very close explains that the sensitivities are very similar (note that the relative variation in the sensitivities for the three frequencies is very small in Figure 6b). Therefore, for sensors based on LC structures where the main target is sensitivity optimization in the vicinity of the REF dielectric constant, thereby sacrificing linearity, the optimum frequency approximately aligns with the resonance frequency. Conversely, if linearity is prioritized over sensitivity, thereby sacrificing sensitivity, then it is important to tune the resonance frequency to *f*_opt_, not to the resonance frequency, *f*_0_, of the sensing resonator.

## 4. Conclusions

In conclusion, it has been demonstrated in this paper that the optimum operating frequency for sensitivity enhancement in reflective-mode phase-variation permittivity sensors based on semi-lumped resonant elements (in particular SIRs, in this work) does not coincide with the frequency where the phase slope is at maximum. Certainly, both frequencies are very similar, and very close to the resonance frequency, for SIRs with ratios *L*/*C* much larger than the squared reference impedance of the ports (corresponding to high-*Q* resonators), but the difference is substantial for moderate or low-*Q* sensing resonators. Nevertheless, it has been demonstrated in this paper that the optimum frequency to boost up the sensitivity in the limit of small perturbations is comprised between the frequency providing maximum phase slope and the resonance frequency. The difference between the frequency of maximum phase slope and the optimum frequency for sensitivity optimization has been attributed to the fact that when a dielectric sample is placed on top of the sensing resonator, the phase response is displaced to the left but distorted. Thus, a significant phase slope is required to achieve a high sensitivity in the limit of small perturbations, but this does not mean that the optimum frequency is the one corresponding to the maximum phase slope, as has been demonstrated. Such frequencies are very close in high-*Q* resonators, and distant in low-*Q* resonators. However, in this latter case, the phase response is quite linear and the difference in the sensitivities (in the limit of small perturbations) when the device operates at the frequency that optimizes the sensitivity and at the frequency of maximum phase slope is not very significant.

## Data Availability

The original contributions presented in this study are included in the article material. Further inquiries can be directed to the corresponding author.
